# Large area and broadband ultra-black absorber using microstructured aluminum doped silicon films

**DOI:** 10.1038/srep42750

**Published:** 2017-02-16

**Authors:** Zhen Liu, Hai Liu, Xiaoyi Wang, Haigui Yang, Jinsong Gao

**Affiliations:** 1 Key Laboratory of Optical System Advanced Manufacturing Technology, Changchun Institute of Optics, Fine Mechanics and Physics, Chinese Academy of Sciences, Changchun 130033, China; 2 University of the Chinese Academy of Sciences, Beijing 100039, China

## Abstract

A large area and broadband ultra-black absorber based on microstructured aluminum (Al) doped silicon (Si) films prepared by a low-cost but very effective approach is presented. The average absorption of the absorber is greater than 99% within the wide range from 350 nm to 2000 nm, and its size reaches to 6 inches. We investigate the fabrication mechanism of the absorber and find that the Al atom doped in silicon improves the formation of the nanocone-like microstructures on the film surface, resulting in a significant decrease in the reflection of incident light. The absorption mechanism is further discussed by experiments and simulated calculations in detail. The results show that the doped Al atoms and Mie resonance formed in the microstructures contribute the broadband super-high absorption.

The functions of surfaces to reduce reflection and enhance absorption over a broadband range are highly attractive in areas from space exploration to consumer electronics, such as optical and optoelectronic devices, blackbody cavity, stray light reduction^1^,^2^,^3^,^4^. Metamaterials are promising candidates for producing absorber^5^,^6^,^7^. However, the absorption of this kind of absorber is limited to a narrow spectral range due to their resonant nature. Therefore, combining several resonator with neighbor spectrum together is an effective method to broaden the absorption spectrum^8^. Silicon (Si) nanostructure, such as nanopillar, nanowire, nanocone arrays, is another kind of absorber which has been widespread investigated due to its broadband absorption compared to metamaterial absorbers. The enhanced high-absorption has been attributed to the following factors, including graded refractive index, multiple light scattering and Mie resonance^9^,^10^,^11^,^12^. It is well known that metallic nanoparticles can improve absorption due to its localized plasmon resonance. Therefore, Si nanostructures combined with metallic nanoparticles to obtain broadband absorbers attract more attentions in recent years^1^,^13^,^14^.

The absorber based on metamaterials can be fabricated by electron beam lithography or focused ion beam milling. Si based nanostructure arrays can be fabricated by electrochemical etching, femtosecond-laser pulses, lithography and metal-assistant chemical etching^15^,^16^,^17^,^18^, but all the fabrication methods mentioned above are costly, time consuming, and only suitable for small areas. To overcome the fabrication limitation, Nan Zhang^19^ *et al*. developed thin-film resonant and nonresonant absorbers using metal-dielectric nanocomposite materials. The basic structure of this absorber is a substrate (glass) coated with metal film followed by a nanocomposite film (from the bottom to the top). The optical characterization can be engineered by controlling the shapes, dimensions of the metallic nanoparticles and their matrix. The absorption over 81% was obtained from 400 nm to 1100 nm. But its absorption is obviously lower than that based on Si nanostructures. Thus large area, low-cost and high performance absorber is strongly desirable.

In this paper, we propose a low-cost but very effective approach to achieve a large-area and broadband ultra-black absorber with an average absorption higher than 99% in a broad wavelength range from 350 nm to 2000 nm. We not only analyze the formation mechanism of nanocone-like structures on SiAl films, but also discuss the super broadband absorption in microstructured SiAl films in detail by a comparison of the intrinsic difference between Si films and SiAl films. Finally, we attempt to explain its origin theoretically by using a numerical simulation.

## Results and Discussion

**Figure 1:**
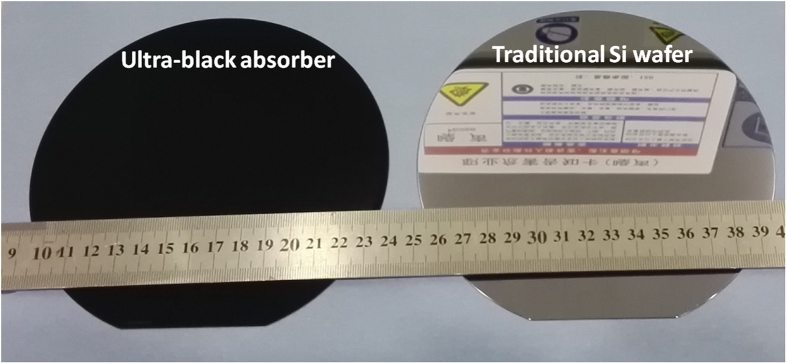
Photo of the absorber and silicon wafer.

**Figure 2:**
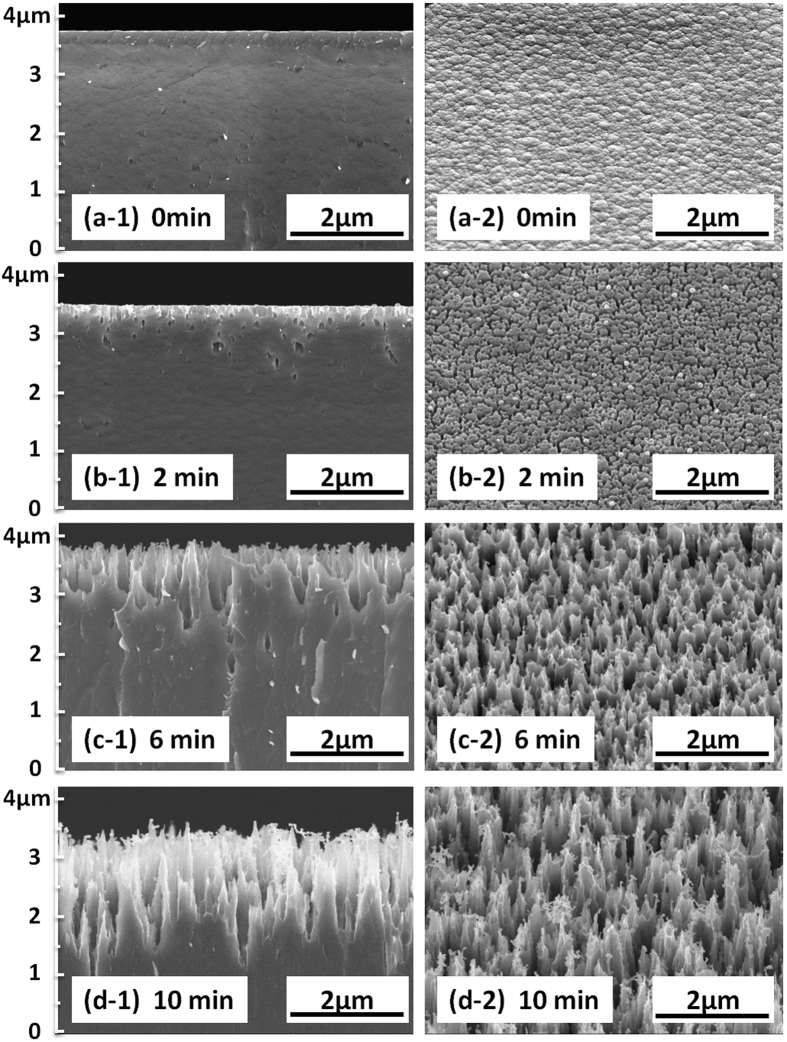
Cross-section and 45° view SEM images of 7 μm-thick SiAl films. (**a**) Without etching, (**b**) etched for 2 min, (**c**) etched for 6 min, and (**d**) etched for 10 min.

**Figure 3:**
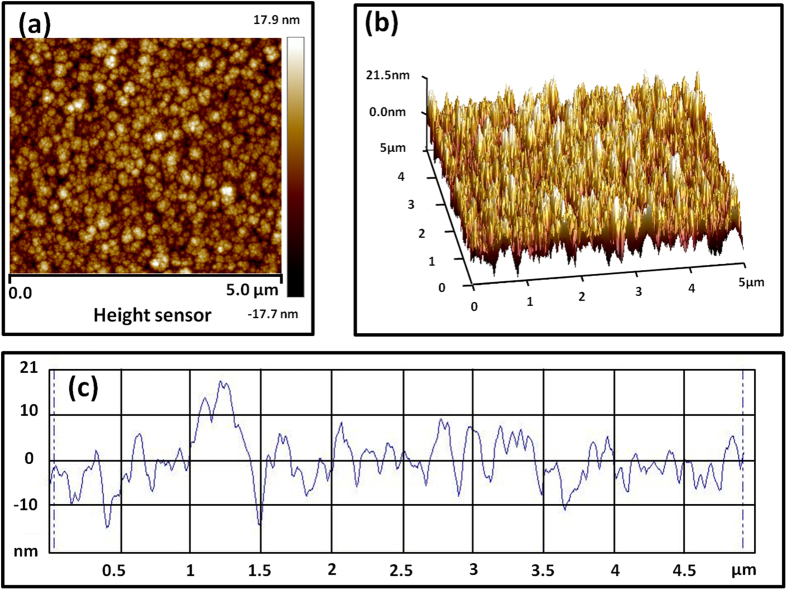
Surface morphology of 7 μm-thick SiAl films. (**a**) Top-view image, (**b**) three-dimensional image, and (**c**) surface fluctuation profile along the white line.

Figure 1 shows a photograph of 6-inch broadband ultra-black absorber, which is fabricated by a deposition of 7 μm-thick SiAl film with an Al concentration around 10% and subsequent 10 min wet-etching in NaOH solutions. As a comparison, a Si wafer without any treatment is also presented. We can see that the fabricated absorber exhibits a very high level of absorption across the visible spectral range, obviously different from the wafer without any treatment. Figure 2 shows both the top-view at 45° angle and cross-section SEM images of 7 μm-thick SiAl films with different wet etching times. For the as grew SiAl film showed in Fig. 2(a), its surface consists of a large amount of grains with size of several ten to hundred nanometers. Further detail of its surface morphology taken with AFM method is present in Fig. 3. It can be concluded from the surface fluctuation profile that the fluctuation from peak to valley around grain boundaries is more than 10 nm, indicating a rough surface as shown in the three-dimensional image. This should be attributed to the grown dynamic process of thin film by physical vapor deposition (sputtering or evaporation). As the film grows it generally exhibits a columnar morphology, and correspondingly its surface roughness gradually increases due to geometrical effects^20^.

After 2 min etching, it is very clear from Fig. 2(b) that the porous structures with a depth of 100–300 nm are formed on SiAl film surface. More importantly, porous etching mainly occurred along the grain boundaries. As etching time increasing, more and more small holes generate and their depth also increases. When the etching time increases to 10 min, the surface microstructures in Fig. 2(d) are developed to a nanocone-like profile from the initial porous one. Moreover, they exhibit a random distribution and an irregular shape with nanometer size. Utilizing the same wet etching method, we also treat pure Si films sputtered on Si substrate. However, we find that no any obvious microstructures are obtained on Si film surface. Therefore, we conclude that the microstructure formation is strongly related to Al atom doping. By a comparison of Fig. 2(a) and (b), it is found that both the shape and size of left SiAl structures after short-time etching are very similar to that of the grains on the as-grown sample surface. This phenomenon suggests that the reaction first occurs around the boundaries. At the preliminary stage of wet-etching, NaOH solutions penetrate SiAl films along the grain boundaries and react with Al atoms around the boundaries. Meanwhile, in NaOH solutions Al potential is higher than Si potential. This potential difference results in local electrochemistry reaction^21^. Al atoms reaction with NaOH solutions produces NaAlO_2_ solved in the solution and releases H_2_ gas while the residual Si atoms cannot react in the solutions. It leads to the porous structures in initial stage of etching. With etching time increasing, the nanocone-like microstructures are finally formed. In another word, it is the existence of Al atoms that boosts the chemical reaction resulting in the formation of nanocone-like microstructures.

**Figure 4:**
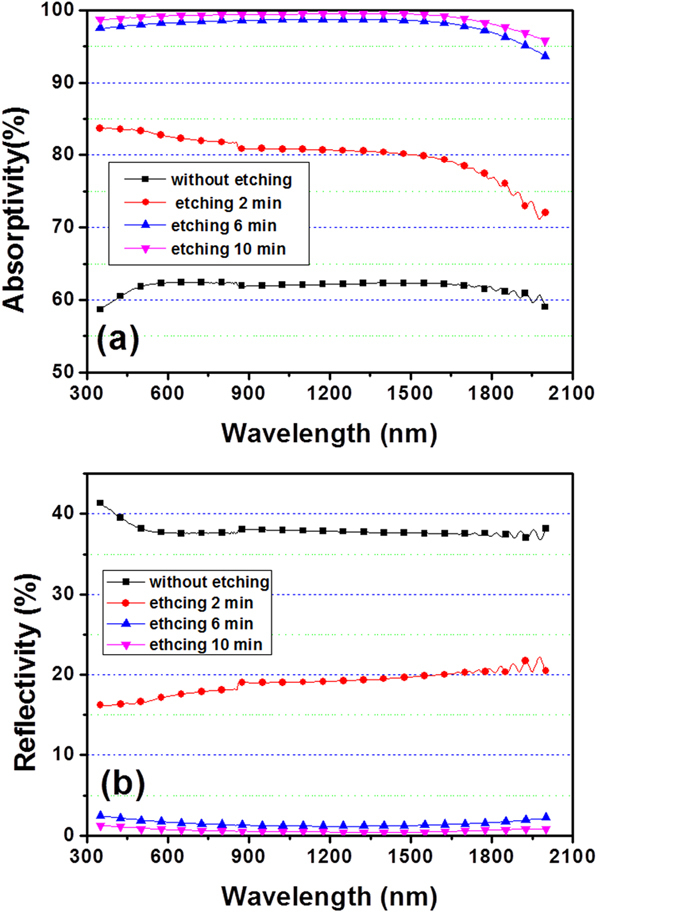
Measured absorption and reflection of silicon absorbers with different etching time.

Figure 4(a) shows the absorption spectra of SiAl films on Si substrate as a function of etching times. After 2 min etching, the average absorption is as high as 80.2% across both the visible and infrared range from 350 nm to 2000 nm. The longer etching time, the higher absorption will become. When the etching time increases to 10 minutes, the average absorption exceeds 99.0% from 350 nm to 2000 nm, and the highest absorption reaches 99.6% at 1380 nm. These results confirm that a broadband ultra-black absorber can be achieved conveniently by wet-etching of SiAl films. It is considered firstly that the surface nanocone-like microstructures in Fig. 2 are one of the dominant contributions to an ultra high absorption. Figure 4(b) shows the reflectance spectra of SiAl films on Si substrate as a function of etching times. The as grown sample has a high reflectance with an average value of approximately 38%. However, it drastically decreases to 19% after only 2 min etching. When prolong the etching time to 10 min, it is clear that the average reflectance is significantly reduced to an extremely low value that lower than 0.7% across both the visible and infrared range from 350 nm to 2000 nm. Obviously the surface with nanocone-like microstructures contributes an excellent antireflective function. It is well known that a textured surface could replace conventional antireflection coating resulting in a low surface reflectance. Some groups utilized lithography and Ag assisted etching to fabricate a low-reflectance textured surface on Si substrate^1^. Some groups reported several lithography-free methods such as self-assembled metal (Ag or Au) nanomask deposition and the subsequent wet or dry etching^1^,^22^,^23^,^24^. Low-reflectance textured surface can be also fabricated by femto-second laser microstructured processing^25^. Significantly different from the methods mentioned above, this paper proposed a novel approach which is simple but very efficient to achieve a low-reflectance microstructured surface.

**Figure 5:**
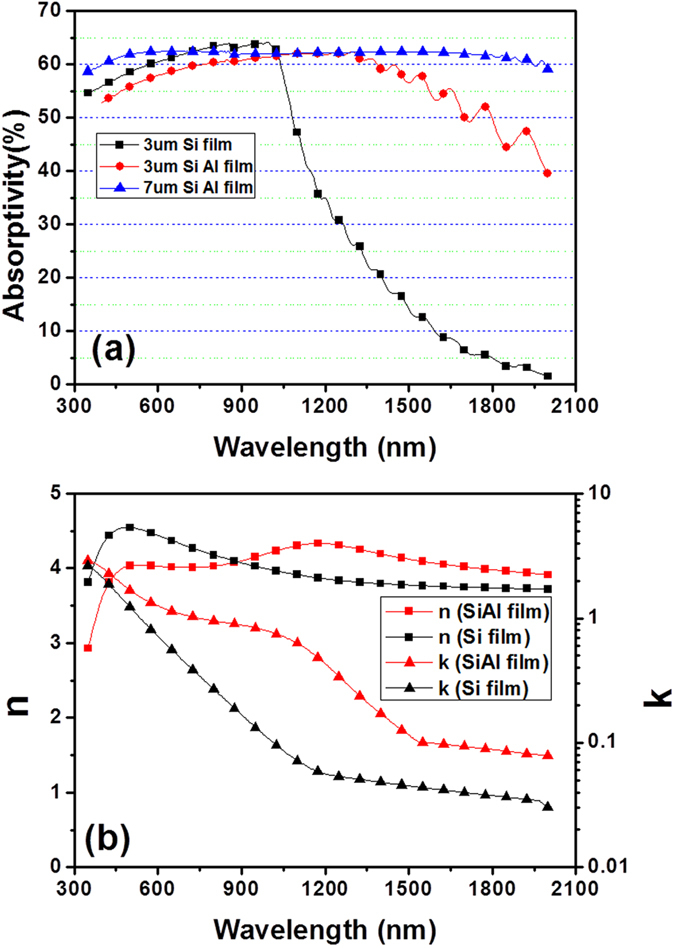
Absorption and optical constants of SiAl and Si films.

Besides the significant suppression of surface reflectance by nanocone-like microstructures, Al atoms doping in SiAl films act as the second key factor to contribute an ultra high absorption. To clarify this, we compare the absorption spectra of pure Si and SiAl films sputtered on Si substrates as showed in Fig. 5(a). Here, samples have no any surface etching treatment. The absorption of Si sputtered films is similar to traditional Si wafer. It has an average absorptance of 60% in the visible region but drastic decreases to 10% in the wavelength longer than 1200 nm due to its large band gap of 1.12 eV. By contrast, SiAl films sputtered on Si substrate exhibit a high absorption behavior in both visible and infrared region, and as SiAl films grow thicker, its absorption further increases especially in the infrared region. The average absorption of 7 μm-thick SiAl films can be up to 62% (350 nm~2000 nm). Figure 5(b) displays a comparison of optical constants between sputtered pure Si and SiAl films, which are extracted by spectroscopic ellipsometry. The main difference between them is that the extinction coefficients (k) of SiAl films are obviously higher than that of pure Si films in the infrared region. This high absorption is due to the free electron oscillation of Al atom with incident light^26^. Therefore, we can conclude that Al atom doping in Si films improves the extinction coefficients and compensates for less absorption of Si in the infrared region, resulting in a broadband high absorption in both visible and infrared region.

**Figure 6:**
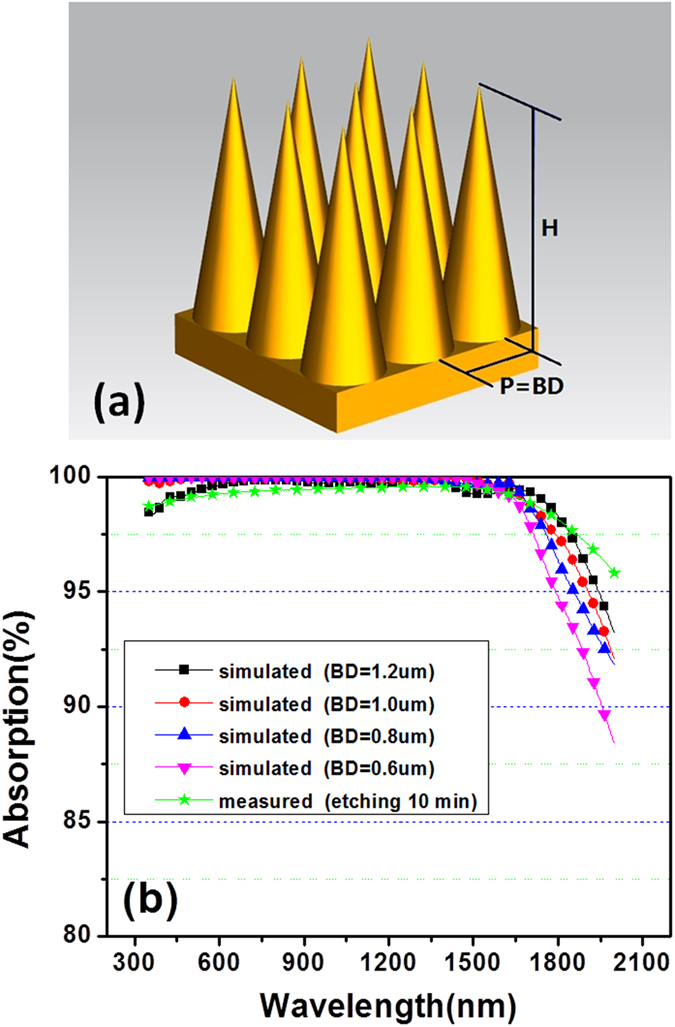
Theoretical model of nanocone-like arrays used in the simulation (**a**), and a comparison of simulated and measured absorption spectra (**b**).

Finally, we simulate the nanocone-like microstructure theoretically by finite difference and time domain (FDTD) algorithm to further clarify its absorption mechanism. Figure 6(a) shows the theoretical model used in the simulation, which is regular and periodic array as a substitute for the irregular microstructures prepared by 10 min etching in Fig. 2(d). SiAl nanocone-like height (H) is set at 2 μm close to that after 10 min etching. The microstructure period (P) is equal to the bottom diameter (BD) of nanocone-like as a variable parameter. Optical constants of SiAl materials used in simulation are from Fig. 5(b). Figure 6(b) shows a comparison of the simulated and measured absorption. All the simulated results exhibit a broadband ultra-high absorption near 100% across the spectral range from 350 nm to 2000 nm. The larger BD size, the higher absorption will become in the longer wavelength range. The simulated absorption is well agreement with the measured. Some small discrepancies between the measured absorption and the simulated should be resulting from the irregularities and defects of the nanocone-like structures. Here it is noted that the theoretical model is regular while the fabricated structures is irregular. By further simulation using a theoretical model with relatively irregular nanocone-like structures, we found that high absorption similar to Fig. 6(b) was also obtained. Thus we considered that the regular model as a substitute for the irregular is reasonable.

**Figure 7:**
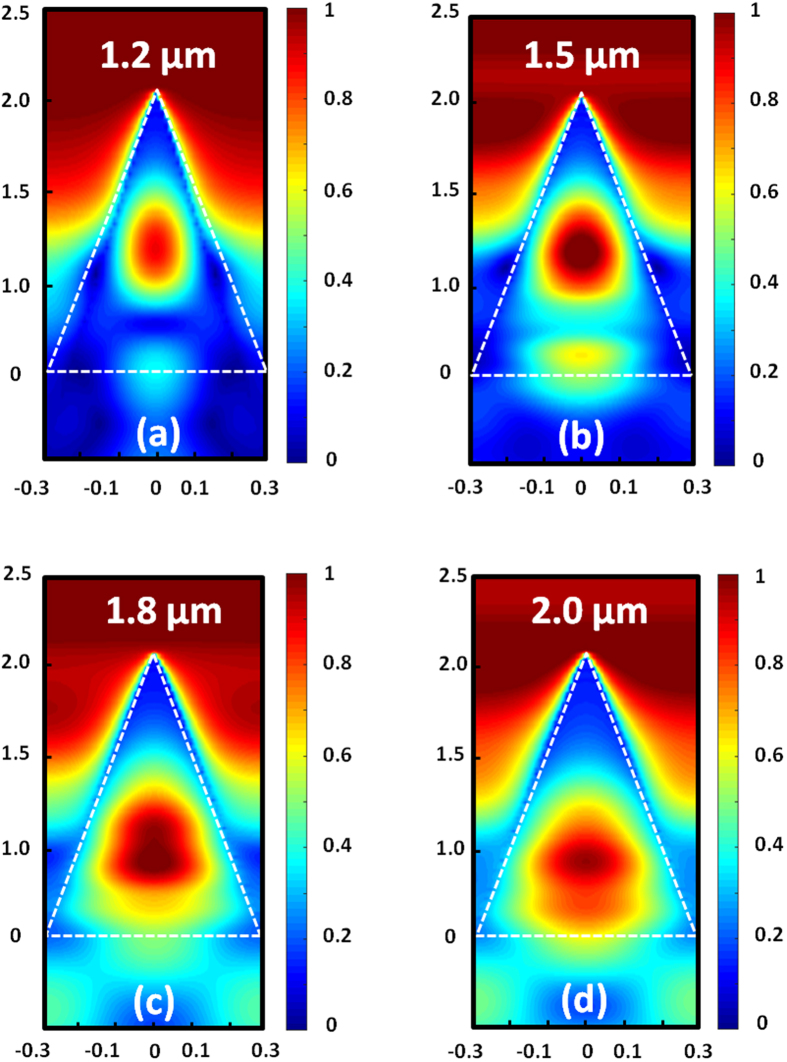
The distributions of electric field (|E|) at different wavelengths. (**a**) 1.2 μm, (**b**) 1.5 μm, (**c**) 1.8 μm, and (**d**) 2.0 μm.

In addition, the distributions of electric field (|E|), as shown in Fig. 7, at various incident wavelengths with TM polarization were simulated. It is obviously that |E| is enhanced dramatically within the nanocone-like structures and different wavelengths will be confined and trapped at different positions in the nanocone-like structures. It can be seen that in the infrared band, the diameter of the trapped position increase with the increasing of the wavelength. The nanocone-like structure exhibits strong absorption across a broadband of wavelength due to the continuously variation of the cross section diameters from bottom to the apex. This phenomenon can be attributed to Mie resonance which enhanced the infrared absorption^12^.

### Fabrication

In this experiment, we fabricate a broadband ultra-black absorber on single-side polished 400 μm-thick (100) -Si substrate with a moderate resistivity (~10 Ω⋅cm). After wet-chemical cleaning, we deposit a SiAl film on Si substrate by a co-sputtering method, where silicon and aluminum is deposited by RF (150 W) and DC (80 W) magnetron sputtering, respectively. The gas flow of Ar is 20 sccm and the pressure of the vacuum chamber is kept at 0.3 Pa. Subsequently, we dip the sample into a NaOH solution for several minutes to produce a microstructural surface by a local electrochemistry reaction induced by Al atom doping. We measure the integrated reflectance (R) and transmittance (T) spectra between 350 nm to 2000 nm in a Lambda-1050 spectrometer equipped with a 160 mm integrating sphere, which is calibrated by a Labsphere Spectralon reflectance standard. Then we extract the integrated absorptance (A) spectra by the formula of A = 1 − R − T. The equipment has a systematic error of reflectivity less than 0.5% in UV/Vis and less than 1% in near infrared. Because of constant usage, the contaminations and scratches will affect the Spectralon’s reflectance, leading to an overestimation of the measured sample reflectance by at least 1% of the measured value in UV/Vis and up to 2% of the measured value in near infrared. The surface microstructure characters are evaluated by scanning electron microscope (SEM) and atomic force microscope (AFM). The metal amount in SiAl films, the film thickness and its optical constants are also determined by energy dispersive X-ray spectroscopy (EDX), profilemeter and spectroscopic ellipsometry, respectively.

## Conclusion

In conclusion, we demonstrated a simple and cost-effective method to produce large-area broadband ultra-black absorber. By SiAl film deposition and subsequent chemical etching, we fabricated the absorber with the size of 6 inches. It exhibits an average absorption higher than 99% within the wide range from 350 nm to 2000 nm. We found that nanocone-like microstructures on the film surface could be fabricated easily by Al atom doping, leading to a significant decrease in the reflection of incident light. In addition, Al atom doping in Si films enhance absorption in the infrared region compared to pure Si films. Theoretical simulation indicated that Mie resonance formed in the microstructures contribute the broadband super-high absorption.

## Additional Information

**How to cite this article:** Liu, Z. *et al*. Large area and broadband ultra-black absorber using microstructured aluminum doped silicon films. *Sci. Rep.* **7**, 42750; doi: 10.1038/srep42750 (2017).

**Publisher's note:** Springer Nature remains neutral with regard to jurisdictional claims in published maps and institutional affiliations.
